# *Streptococcus pneumoniae* and *Haemophilus influenzae* type b carriage in Chinese children aged 12–18 months in Shanghai, China: a cross-sectional study

**DOI:** 10.1186/s12879-016-1485-3

**Published:** 2016-04-14

**Authors:** Jiayu Hu, Xiaodong Sun, Zhuoying Huang, Abram L. Wagner, Bradley Carlson, Jianping Yang, Suwen Tang, Yunyi Li, Matthew L. Boulton, Zhengan Yuan

**Affiliations:** Shanghai Centers for Disease Control and Prevention, 1380 Zhongshan West Road, Shanghai, 200336 China; Department of Epidemiology, University of Michigan, 1415 Washington Heights, SPH II, Ann Arbor, MI 48109 USA

**Keywords:** *Streptococcus pneumoniae*, *Haemophilus influenzae* type b, Seroprevalence, Antimicrobial susceptibility, China

## Abstract

**Background:**

The bacteria *Streptococcus pneumoniae* (pneumococcus) and *Haemophilus influenzae* type b (Hib) are leading causes of childhood pneumonia and meningitis and are major contributors to worldwide mortality in children younger than 5 years of age. Asymptomatic nasopharyngeal carriage of pneumococcus and Hib was determined for healthy children in Shanghai in 2009.

**Methods:**

Children from 5 immunization clinics were enrolled in this study. Specimens from the nasopharynx were collected and cultured in Columbia and chocolate agar to identify pneumococcal and Hib carriage. Pneumococcal specimens were serotyped with the Neufeld test, and antibiotic resistance for pneumococcal and Hib specimens used the E-test method. Significance of risk factors for carriage was assessed through chi-square tests.

**Results:**

Among 614 children, 16.6 % had pneumococcal carriage and 8.0 % Hib carriage. The predominant serotype of pneumococcus that was isolated was 19 F (52.9 %); serotype coverage was 68.6 % for both 7-valent pneumococcal conjugate vaccine (PCV) and PCV-10, and 82.3 % for PCV-13. Household residency and father’s education were both significantly related to pneumococcal and Hib carriage. The majority of *S. pneumoniae* isolates were sensitive to most antimicrobials but there were high levels of resistance to azithromycin (51.0 %) and erythromycin (51.0 %). *Haemophilus influenzae* isolates were sensitive to almost all antimicrobials tested although 12.2 % of isolates were resistant to ampicillin.

**Conclusions:**

The pneumococcal and Hib vaccines require payment, and the children with the highest burden of disease may not be receiving these vaccines. Moreover, the presence of high antibiotic susceptibility towards pneumococcus, and to a lesser extent towards Hib, underscores the need for preventive protection against these diseases. Public funding of pneumococcal and Hib vaccines would be one mechanism to increase uptake of these vaccines.

## Background

The bacteria *Streptococcus pneumoniae* (pneumococcus) and *Haemophilus influenzae* type b (Hib) are leading causes of childhood pneumonia and meningitis and are major contributors to worldwide mortality in children younger than 5 years of age. In 2011, an estimated 411,000 deaths occurred worldwide due to pneumococcal pneumonia and 197,000 due to Hib pneumonia [1]. In China, it is estimated that there are 261,000 cases and 11,000 deaths each year in children under 5 years of age due to pneumococcal pneumonia and meningitis [2], and 19,000 childhood deaths result from Hib infection [3].

Pneumococcus and Hib colonize the upper airways. Asymptomatic carriers can still transmit to other individuals, with disease resulting if the pathogens descend the airway into the lower respiratory tract [4, 5]. Over 90 different serotypes of pneumococcus have been found, and vaccinations need to strike a reasonable balance between the financial cost against the value of including more serotypes known to cause disease [6]. In contrast to the serotypic diversity with pneumococcus, Hib is a much more predominant cause of disease than non-type b *H. influenzae* [7].

The Hib conjugate vaccine has been available since 1987 in the US [7] and 2000 in China [8], and a pneumococcal conjugate vaccine (PCV) was licensed in the US in 2000 [9] and introduced to China in 2008 [10], although it was taken off the market in China in 2014 [11]. Neither the Hib vaccine nor PCV are included on the publically-funded Expanded Program on Immunization (EPI) in China, but they are available at immunization clinics for a fee. The Hib vaccine costs $13–$17 whereas PCV is much more expensive ($127) [10]. As a result coverage of Hib vaccine is much higher than PCV (50.9 % versus 11.4 % in Shanghai); moreover, although infants as young as 6 weeks are eligible for Hib vaccine and PCV administration, vaccination is typically delayed until after 1 year of age. Coverage at 12 months is around 20 % for Hib vaccine and <5 % for PCV and at 24 months, coverage is approximately 40 % for Hib vaccine and 5 % for PCV [12].

The first PCV protected against 7 serotypes of pneumococcus (PCV-7), but more recently, 10-valent (PCV-10) and 13-valent (PCV-13) vaccines have been licensed [6], and a 15-valent vaccine (PCV-15) is under development [13]. The World Health Organization (WHO) recommends that countries choose a PCV depending on the distribution of serotypes within their population, along with vaccine supply and cost-effectiveness considerations [6]. Two studies in China have previously evaluated the coverage between PCVs and serotypes in the population. A study of clinical isolates from 8 cities in China during 2005 and 2006 found coverage of PCV-7 was 62.6 %, PCV-10 was 64.8 %, and PCV-13 was 79.6 % [14]. Similarly, a study of clinical isolates from 2006 to 2008 found coverage of 60.3, 66.7 and 87.8 % for the 3 vaccines, respectively [15]. Because the distribution of pneumococcal serotypes may vary geographically, however, these findings may not be representative of potential coverage from vaccines in all areas.

Few studies have been published from China in the English literature looking at Hib and pneumococcal carriage. This information is important for modeling the burden of disease and underscoring the importance of vaccination, particularly in a country with low coverage of Hib and pneumococcal vaccines but with widespread resistance to antibiotics [16, 17]. In this study, we calculate the prevalence of nasopharyngeal carriage of Hib and pneumococcus (including specific pneumococcal serotypes), describe the prevalence of antimicrobial resistance in children with carriage, and examine risk factors for nasopharyngeal colonization with pneumococcus or Hib.

## Methods

In 2009, April and May for spring, October and November for autumn, healthy children aged 12–18 months were recruited from a total of 5 immunization clinics in Shanghai. We purposely sampled in both the spring and autumn in order to determine if there was a seasonal fluctuation in carriage (although this has not been borne out in previous studies in the UK [18] or Italy [19]). These clinics purposefully selected from 3 districts (Huangpu, Xuhui, and Pudong) to represent different areas of the city. For 1 or 2 days, research staff from the municipal Centers for Disease Control and Prevention (CDC) and district CDCs attended these clinics, and enrolled children between 12 and 18 months who attended the clinic on those days. Children were excluded for the following reasons: 1) antibiotic use within 15 days of enrollment; 2) congenital anomalies of the nasopharynx; 3) existence of a long-term infectious disease (such as chronic otitis media, or chronic sinusitis); 4) previous immunization for pneumococcal disease; 5) coagolopathy; or 6) body temperature over 38 °C at time of enrollment.

Parents of the children were asked about their residency status (local or Shanghai residency vs non-local residency), their household monthly income, daycare attendance, household size, number of children, highest educational level attainment of father and mother, smoking in the household,, if child was breastfed, child history of serious disease, and number of doses of Hib vaccine the child had received.

Research staff obtained a nasopharyngeal sample using one infant-sized calcium alginate swab (Thermo Fisher Scientific, Hampton, NH, USA) for each child. These specimens were streaked on Columbia agar and chocolate agar immediately upon collection, then transported in 12 h to the Shanghai Municipal CDC for culturing and preliminary pathogen identification. The swabs were streaked directly onto the plates. Positive isolates were stored at −70 °C, then transported to a centralized laboratory in Beijing for pathogen confirmation, pneumococcal serotyping, and antibiotic resistance testing. The site of specimen collection (nasopharynx), swab material (calcium alginate fiber), storage temperature (−70 °C), and choice of agar (5 % sheep blood) followed standard methods [20].

### Isolate confirmation

The specimens streaked on the Columbia and chocolate agar were incubated at 37 °C in 5 % CO_2_-enriched atmosphere for 24–48 h. Identification of pneumococcal colonies was based on the following conventional microbiological methods: colony morphology, growth on Columbia agar, susceptibility to optochin, and bile solubility. Colony morphology, growth on chocolate agar, and the X + V factor were requirements for identification of *H. influenzae* colonies. For both pneumococcus and Hib, a diameter in the inhibition zone >7 mm was recorded as a positive result. *S. pneumoniae* isolates were serotyped by the Neufeld (Quellung) reaction, by inoculating a broth with culture from the agar plate, and then mixing a selection from the broth with antiserum and determining if a reaction occurred.

### Antimicrobial susceptibility tests

Minimum inhibitory concentrations (MICs) of anti-microbial agents were determined by E-test method. The pneumococcal and Hib isolates were tested for susceptibility to azithromycin, cefuroxime, ceftriaxone, levofloxacin, and moxifloxacin. Pneumococcal isolates were additionally tested for resistance to amoxicillin/clavulanic acid, erythromycin, and penicillin. *H. influenzae* isolates were tested for susceptibility to ampicillin. Standard references were included for quality control.

### Statistical analysis

Descriptive statistics were used to illustrate the prevalence of Hib and pneumococcal carriage, antibiotic resistance, *S. pneumoniae* serotypes, season of enrollment, and other demographic characteristics of the enrolled children. For bivariate analyses, the Pearson chi-square test was used to test the association between dichotomous demographic factors and 1) carriage of *S. pneumoniae* and 2) carriage of Hib. The Cochran-Armitage Trend Test was used to compute *P*-values for ordinal variables (age of child, age of mother at childbirth, urbanicity, household income, size of house, mother’s education, father’s education, feeding pattern, and Hib vaccine history). Exact tests were used if any cell counts were ≤5. Correction for multiple testing was done through the sequential Holm-Bonferroni Method.

For the multivariable analysis, the explanatory variables input into the logistic regression model were either related due to (1) a priori considerations (age of child), (2) selection criteria (season, urbanicity), or (3) variables found to be significant (*P*-value <0.05) in the bivariate analysis. Mother’s education level and father’s education level were not placed in the same model together because of concerns about multicollinearity. Analyses were performed in SAS version 9.3 (SAS Institute, Inc, Cary, NC, USA). *P*-value correction for multiple testing was computed in R version 3.0.3 (R Foundation for Statistical Computing, Vienna, Austria).

## Results

In this study, 6 parents refused to participate, leaving a total of 614 children aged 12 to 18 months enrolled: 308 in the spring and 306 in the autumn. Table 1 highlights the demographic and carrier state risk factors; 338 were male, and 160 were local residents, whereas 454 were non-local children whose family had relocated to Shanghai from another province. Children with PCV already administered were excluded from participating in the study, but 80.7 % of children had received at least one dose of Hib vaccine. No child in the study attended a daycare.

**Table 1:** Prevalence of nasopharyngeal carriage of pneumococcus and *Haemophilus influenzae* type b (Hib) and demographic and medical correlates among children in Shanghai, 2009

	Count	Pneumococcal carriage (%)	*P*-value^a^	Hib carriage (%)	*P*-value^a^
Overall	614	102 (16.6 %)		49 (8.0 %)	
Season of enrollment			0.0780		1.0000
Autumn	306	38 (12.4 %)		23 (7.5 %)	
Spring	308	64 (20.8 %)		26 (8.4 %)	
Sex			1.0000		1.0000
Male	338	60 (17.8 %)		30 (8.9 %)	
Female	276	42 (15.2 %)		19 (6.9 %)	
Age of child			0.1530		1.0000
12 months	170	35 (20.6 %)		14 (8.2 %)	
13–14 months	184	35 (19.0 %)		16 (8.7 %)	
15–16 months	158	20 (12.7 %)		12 (7.6 %)	
17–18 months	102	12 (11.8 %)		7 (6.9 %)	
Age of mother at childbirth			0.3984		1.0000
18–24 years	179	35 (19.6 %)		18 (10.1 %)	
25–29 years	257	44 (17.1 %)		18 (7.0 %)	
30–34 years	123	18 (14.6 %)		9 (7.3 %)	
35–44 years	55	5 (9.1 %)		4 (7.3 %)	
Household residency			0.0009		0.0008
Local	160	11 (6.9 %)		2 (1.3 %)	
Non-local	454	91 (20.0 %)		47 (10.4 %)	
Urbanicity			0.1530		0.0077
Urban	422	62 (14.7 %)		25 (5.9 %)	
Suburban	126	22 (17.5 %)		12 (9.5 %)	
Rural	66	18 (27.3 %)		12 (18.2 %)	
Household income			0.1166		0.0490
<2000 yuan ($292)	232	47 (20.3 %)		26 (11.2 %)	
2000–4999 yuan ($292–$730)	286	47 (16.4 %)		21 (7.3 %)	
≥5000 yuan ($731)	96	8 (8.3 %)		2 (2.1 %)	
Size of house			0.2163		0.0008
<18 m^2^	145	32 (22.1 %)		17 (11.7 %)	
18–29 m^2^	120	20 (16.7 %)		19 (15.8 %)	
30–69 m^2^	156	25 (16.0 %)		8 (5.1 %)	
≥70 m^2^	193	25 (13.0 %)		5 (2.6 %)	
Siblings			0.1530		0.0999
No	407	57 (14.0 %)		24 (5.9 %)	
Yes	207	45 (21.7 %)		25 (12.1 %)	
Mother’s education			0.0650		0.0008
Primary school or less	73	18 (24.7 %)		9 (12.3 %)	
Junior high	286	54 (18.9 %)		32 (11.2 %)	
High school/vocational	134	16 (11.9 %)		8 (6.0 %)	
College or more	121	14 (11.6 %)		0 (0.0 %)	
Father’s education			0.0406		0.0003
Primary school or less	23	4 (17.4 %)		4 (17.4 %)	
Junior high	299	65 (21.7 %)		34 (11.4 %)	
High school/vocational	135	16 (11.9 %)		10 (7.4 %)	
College or more	157	17 (10.8 %)		1 (0.6 %)	
Smoking in home			1.0000		1.0000
No	288	47 (16.3 %)		27 (9.4 %)	
Yes	326	55 (16.9 %)		22 (6.7 %)	
Feeding pattern (first 6 months)			0.8612		0.3392
Exclusive breastfeeding	317	58 (18.3 %)		35 (11.0 %)	
Breastfeeding and formula	207	32 (15.5 %)		7 (3.4 %)	
Only formula	90	12 (13.3 %)		7 (7.8 %)	
Any history of disease			0.6870		0.8154
No	123	26 (21.1 %)		14 (11.4 %)	
Yes	491	76 (15.5 %)		35 (7.1 %)	
Hib vaccine history			1.0000		0.3392
None	71	14 (19.7 %)		9 (12.7 %)	
1 dose	146	22 (15.1 %)		14 (9.6 %)	
2 doses	148	24 (16.2 %)		12 (8.1 %)	
3 doses	182	31 (17.0 %)		10 (5.5 %)	
4 doses	67	11 (16.4 %)		4 (6.0 %)	

^a^From chi-square test, except for age of child, age of mother at childbirth, urbanicity, household income, size of house, mother’s education, father’s education, feeding pattern, and Hib vaccine history, which were from Cochran-Armitage Trend Test. Exact tests were used if cell counts were ≤5. *P*-values were corrected for multiple testing through the sequential Holm-Bonferroni Method

Overall, 137 (22.3 %) of participants were carriers of one or more pathogen. More children were carriers of pneumococcus (16.6 %) than Hib (8.0 %). Household residency and father’s education were the two significant risk factors for pneumococcal carriage: more non-locals (20.0 %) than locals (6.9 %) were carriers (*P* = 0.0009), and children having a father with less education were more likely to be carriers (*P* = 0.0406). For Hib carriage, in addition to household residency and father’s education, urbanicity, household income, size of house, and mother’s education were significant risk factors. Urban dwellers had lower carriage (5.9 %) than those in suburban (9.5 %) or rural areas (18.2 %) (*P* = 0.0077), and having greater income and a larger house were protective against Hib carriage. Hib vaccination was not associated Hib carriage status (*P* = 0.3392).

The most common pneumococcal serotype was 19 F, which was in 54 children. In descending order of prevalence, the other isolated serotypes present in the sample were 19A (*n* = 8), 6A (*n* = 6), 6B (*n* = 5), 6A/6B cross-reactive (*n* = 5), 23 F (*n* = 4), 15 (*n* = 4), 14 (*n* = 2), and 1 each for 17 and 22Twelve specimens were untypeable. The prevalences we found correspond to a coverage of 68.6 % (70) for PCV-7 and PCV-10, 82.3 % (84) for PCV-13, and 83.3 % (85) for PCV-15.

Table 2 shows the frequency of antimicrobial resistance for each pathogen. All 102 pneumococcal isolates were sensitive to levofloxacin and moxifloxacin. Highest levels of resistance were to azithromycin (51.0 %) and erythromycin (51.0 %). Most Hib isolates were sensitive to all antibiotics, except 12.2 % were resistant to ampicillin.

**Table 2:** Antibiotic Resistance among *S. pneumoniae,* and *H. Influenzae type b* isolates in 614 Shanghai children, 2009

Antibiotic	*S. pneumoniae* (*n* = 102)			*H. influenzae* type b (*n* = 49)		
	Sensitive	Intermediary	Resistant	Sensitive	Intermediary	Resistant
Amoxicillin/Clavulanic Acid	96 (94.1 %)	5 (4.9 %)	1 (1.0 %)	–	–	–
Ampicillin	–	–	–	43 (87.8 %)	0	6 (12.2 %)
Azithromycin	50 (49.0 %)	0	52 (51.0 %)	49 (100.0 %)	0	0
Cefuroxime	82 (80.4 %)	4 (3.9 %)	16 (15.7 %)	49 (100.0 %)	0	0
Ceftriaxone	102 (96.1 %)	3 (2.9 %)	1 (1.0 %)	49 (100.0 %)	0	0
Erythromycin	50 (49.0 %)	0	52 (51.0 %)	–	–	–
Levofloxacin	102 (100.0 %)	0	0	49 (100.0 %)	0	0
Moxifloxacin	102 (100.0 %)	0	0	49 (100.0 %)	0	0
Penicillin	96 (94.1 %)	6 (5.9 %)	0	–	–	–

Results of a multivariable analysis are shown in Table 3. Pneumococcal carriage was significantly lower in the spring compared to autumn (odds ratio (OR) = 0.46, 95 % confidence interval (CI) = 0.27, 0.78). Locals had 0.30 times the odds of pneumococcal carriage compared to non-locals (95 % CI = 0.13, 0.69). No significant predictors of Hib carriage were found; a more parsimonious model of Hib carriage, only including the covariates from the pneumococcal carriage model, also revealed no statistically significant predictors (results not shown).

**Table 3:** Odds ratios and 95 % confidence intervals for risk factors of nasopharyngeal carriage of pneumococcus or *Haemophilus influenzae* type b (Hib) among 614 children in Shanghai, 2009

	Pneumococcal carriage	*P*-value^a^	Hib carriage	*P*-value^a^
Spring vs autumn	0.46 (0.27, 0.78)	0.0044	0.78 (0.37, 1.64)	0.5166
Age of child		0.4703		0.9533
12 months	1.46 (0.69, 3.09)		0.95 (0.34, 2.65)	
13–14 months	1.34 (0.64, 2.84)		1.08 (0.39, 2.94)	
15–16 months	0.94 (0.42, 2.09)		1.21 (0.43, 3.40)	
17–18 months	ref		ref	
Local vs non-local residency	0.30 (0.13, 0.69)	0.0048	0.45 (0.09, 2.32)	0.3388
Urbanicity		0.1773		0.1067
Urban	0.66 (0.34, 1.25)		0.43 (0.19, 0.97)	
Suburban	1.06 (0.46, 2.44)		0.63 (0.21, 1.85)	
Rural	ref		ref	
Father’s education		0.4037		0.4163
Primary school or less	0.79 (0.21, 2.90)		8.53 (0.70, 103.52)	
Junior high	1.10 (0.52, 2.34)		5.61 (0.60, 52.28)	
High school/vocational	0.66 (0.29, 1.52)		5.29 (0.58, 48.43)	
College or more	ref		ref	
Household income				0.5869
<2000 yuan			2.21 (0.48, 10.18)	
2000–4999 yuan			2.19 (0.47, 10.18)	
≥5000 yuan			ref	
Size of house				0.1010
<18 m^2^			2.06 (0.67, 6.32)	
18–29 m^2^			2.96 (0.99, 8.85)	
30–69 m^2^			1.17 (0.35, 3.88)	
≥70 m^2^			ref	
≥1 dose Hib vaccine vs no doses			0.55 (0.24, 1.27)	0.1607

^a^ *P*-value from Wald Chi-square test of Type 3 Analysis

## Discussion

In a study of a group of otherwise healthy children 12–18 months of age attending immunization clinics in Shanghai, we found a substantial number had asymptomatic nasopharyngeal colonization with either pneumococcus (16.6 %) or Hib (8.0 %). Carriage of *Haemophilus influenzae* type b in Shanghai was higher compared to concurrent studies taking place in Dongguan and Beijing [21, 22]. Pneumococcal carriage was lower in this study than that in Dongguan [21], but higher than that in Beijing [22]. The rate of pneumococcal carriage we found is slightly lower than what has been found in the United States (29 %) and Australia (26 %) prior to widespread pneumococcal vaccination [23, 24], and it is much lower than what has been found in other developing countries before vaccination introduction; for example almost all infants are colonized with pneumococcus in Papua New Guinea and The Gambia [25]. Low carriage of these pathogens among persons in Shanghai may result from high levels of antibiotic overuse [16], and Hib carriage might be lower than pneumococcal carriage because children with a history of PCV administration were excluded, whereas most children in the study had received at least one dose of Hib vaccine. There may be a substantial reduction in carriage due to Hib vaccine coverage in the population; a study in The Gambia found nasopharyngeal carriage decreased from 12 to 0.5 % after the Hib vaccine was introduced [26].

It has been hypothesized that crowding, having more children in the family, and exposure to smoking are reasons why carriage is higher in developing countries than developed countries [25]. We found an inverse association between household size and Hib (but not pneumococcal) carriage, and carriage was higher (but not significantly so) for children with siblings compared to single children, indicating possible effects from human crowding. However, families in this study showed similar characteristics to families throughout China—they had only child, and typically family members (like grandparents) take care of children during the day instead of parents utilizing community daycare facilities. Accordingly, risk factors (like large families and daycare use) common in other places throughout the world may have a low prevalence in China and may not be as important for describing the overall distribution of nasopharyngeal carriage in the population.

Approximately two-thirds of the pneumococcal isolates identified are included in the current PCV-7 formulation, and there is increased coverage, to about four-fifths, with PCV-13. In contrast, the additional serotypes in PCV-10 and PCV-15, which build upon PCV-7 and PCV-13, respectively, do not appear to be highly prevalent in Shanghai children. This suggests that PCV-13 is an appropriately comprehensive vaccine for use in Shanghai.

Antibiotic resistance tests are important to ensure that the clinical treatment is effective. We encountered high resistance to erythromycin and azithromycin among pneumococcal isolates in this study, and previous studies have even higher levels of antibiotic resistance to erythromycin and penicillin [27]. Antibiotic resistance in the Hib isolates was lower than in the pneumococcal isolates, but the relatively high resistance rate for Hib (12.2 %) is concerning. Hib and pneumococcus can cause a similar spectrum of invasive disease—pneumonia, meningitis, or septicemia; and doctors in China often do not identify the causative agent of disease or assess for antimicrobial susceptibility before prescribing a course of treatment. High antibiotic resistance in the population can therefore prevent a child from receiving an effective treatment. As Hib and pneumococcal vaccination coverage increases in China, sustained laboratory investigations of antibiotic resistance will be necessary to ensure that the standard of care for invasive disease is effective. Because penicillin sensitivity was still quite high in this population, it may be the antibiotic of choice for physicians in Shanghai treating pneumococcal disease.

This study has several limitations. We collected specimens from asymptomatic children, and, theoretically, the pneumococcal serotypes in healthy children may differ from those with invasive pneumococcal disease. However, previous studies in China have found some concordance between the serotype distribution in invasive and noninvasive cases [14]. We also did not test an individual child for multiple carriage of pneumococcal serotypes, although previous studies have found a large proportion of children may be colonized in this manner [28]. Additionally, our small sample size may limit our ability to identify important risk factors for pneumococcal and Hib colonization. Lastly, it is difficult to compare the distribution of risk factors for Hib carriage to those for pneumococcal carriage because we did not exclude participants based on Hib vaccination, and Hib vaccine coverage may be high enough in Shanghai to effectively lower Hib carriage [26].

## Conclusions

We found a substantial proportion of children with asymptomatic pneumococcal and Hib colonization. In recent years, there have been for-fee vaccines available in China to protect against pneumococcal and Hib disease. Because carriage of these bacteria was higher in more disadvantaged children, indicating a higher burden of disease in these children, these vaccines may not be reaching the children who have the highest burden of disease. Moreover, the presence of high antibiotic susceptibility towards pneumococcus, and to a lesser extent towards Hib, underscores the need for preventive protection against these diseases. Public funding of pneumococcal and Hib vaccines would be one mechanism to increase uptake of these vaccines.

## Ethics approval and consent to participate

The Shanghai CDC Ethics Committee approved this study. Informed consent was obtained from each child’s parents prior to participation.

## Consent for publication

Not applicable.

## Availability of data and materials

Please contact the corresponding author for details about the data. Data access is subject to approval from the Shanghai CDC.

## Abbreviations

CDC: Centers for Disease Control and Prevention
EPI: Expanded Program on Immunization
Hib: *Haemophilus influenzae* type b
MICs: minimum inhibitory concentrations
PCV: pneumococcal conjugate vaccine
Pneumococcus: *Streptococcus pneumonia*
WHO: the World Health Organization
